# Homocysteine level at the acute stage of ischemic stroke as a biomarker of poststroke depression: A systematic review and meta-analysis

**DOI:** 10.3389/fpsyt.2022.1016700

**Published:** 2023-02-13

**Authors:** Yanling Liang, Xiangqun Shi, Lue Chen, Yongxin Li, Jianping Zhong

**Affiliations:** Department of Neurology, Shunde Hospital, Southern Medical University (The First People's Hospital of Shunde), Foshan, China

**Keywords:** poststroke depression, homocysteine, risk factor, meta-analysis, acute ischemic stroke

## Abstract

**Background:**

Studies on the association of homocysteine level with poststroke depression (PSD) have yielded conflicting results. This systematic review and meta-analysis aimed to evaluate the elevated homocysteine level at the acute stage of ischemic stroke in predicting PSD.

**Methods:**

Two authors systematically searched articles indexed in PubMed and Embase databases up to 31 January 2022. Studies evaluating the association of homocysteine level with the development of PSD in patients with acute ischemic stroke were selected.

**Results:**

A total of 10 studies involving 2,907 patients were identified. The pooled adjusted odds ratio (OR) of PSD was 3.72 [95% confidence intervals (CI) 2.03–6.81] for the top vs. bottom homocysteine level. The value of elevated homocysteine level in predicting PSD was stronger in ≥6-month follow-up (OR 4.81; 95% CI 3.12–7.43) than those in ≤ 3-month follow-up subgroup (OR 3.20; 95% CI 1.29–7.91). Moreover, a per unit increase in homocysteine level conferred a 7% higher risk of PSD.

**Conclusion:**

Elevated homocysteine level in the acute stage of ischemic stroke may be an independent predictor of PSD.

## Introduction

Stroke remains a global public health burden resulting in substantial mortality and morbidity (1). Neuropsychiatric disorders represent the common consequence associated with stroke (2). Poststroke depression is the most frequent neuropsychiatric disorder (3, 4). Approximately, over 30% of survivors suffered from poststroke depression at the early or late stage of stroke (5). Poststroke depression has been linked with cognitive impairments (6), functional independence (7), recurrent vascular events (8), and worse survival (9). Considering its negative impacts, the identification of effective biomarkers for predicting poststroke depression is of particular importance.

Homocysteine is an amino acid produced by chemically altering adenosine. The elevated blood level of homocysteine has been identified as an independent risk factor for stroke (10). Moreover, increased homocysteine levels can also be recognized as a predictor of adverse outcomes in patients with acute ischemic stroke (11). Interestingly, homocysteine level was higher in individuals with depression than in healthy controls (12). However, available studies regarding the association of homocysteine level with depression after stroke have yielded conflicting results (13–18). Nevertheless, the findings of these studies were established on a small number of patients.

Currently, no previous meta-analysis has yet investigated the association between homocysteine level and the development of poststroke depression. To address this knowledge gap, we conducted this systematic review and meta-analysis to evaluate the utility of elevated homocysteine levels at the acute stage in predicting poststroke depression among ischemic stroke survivors.

## Methods

### Search strategy

This systematic review and meta-analysis were conducted and reported according to the checklist of Preferred Reporting Items for Systematic Reviews and Meta-Analyses statement. Two authors systematically searched articles indexed in PubMed and Embase databases up to 31 January 2022. Search keywords included the following terms in combination: “depression” OR “poststroke depression” OR “post-stroke depression” AND “stroke” AND “homocysteine” OR “hyperhomocysteinemia.” We also manually searched the reference lists of each related article to find any possible missing studies.

### Study selection

The inclusion criteria were as follow: (1) participants with a diagnosis of ischemic or hemorrhage stroke, (2) blood homocysteine level at the acute stage as exposure, (3) depression diagnosed by a valid instrument after stroke as an outcome measure, (4) provided the multivariate-adjusted relative risk of poststroke depression for the top vs. bottom homocysteine level or per unit increase in homocysteine level, and (5) study design: cohort study or *post hoc* analysis of clinical trials. The exclusion criteria included: (1) patients with a history of depression before stroke onset, (2) case-control as study design, and (3) poststroke depression was diagnosed <1 month.

### Data extraction and risk of bias

The data collected from the eligible study included: the first author's name, publication year, year of publication, type of stroke, study design, patients' number, percentage of the male gender, age of patients, tool and time of depression assessment, the prevalence of poststroke depression, the fully adjusted relative risk of poststroke depression, and adjusted covariates. The risk of bias in included studies was evaluated using the Newcastle–Ottawa Scale (NOS) for the cohort studies (19). Studies with seven points or more were defined as low risk of bias. The discrepancy between the two authors was resolved by discussion.

### Statistical analysis

Meta-analyses were carried out using STATA 12.0 (Stata Corp LP, College Station). The association of elevated homocysteine level with poststroke depression was expressed by pooling fully adjusted odds ratio (OR) with 95% confidence intervals (CI) for the top vs. bottom homocysteine level or per unit increase in homocysteine level. Heterogeneity across studies was measured using the *I*^2^ statistic (*I*^2^ > 50% indicating significance) and Cochran's *Q*-test (*p* < 0.10 indicating significance). The selection of a fixed-effect model or random-effect model was based on the without or with significant heterogeneity. Publication bias was evaluated by Begg's test (20) and Egger's test (21). Leave-one-out sensitivity analysis was performed to investigate the reliability of the pooling risk summary. Subgroup analyses were conducted according to the time of poststroke depression assessment (≤ 3 vs. ≥6 months).

## Results

### Search results and studies characteristics

**Figure 1:**
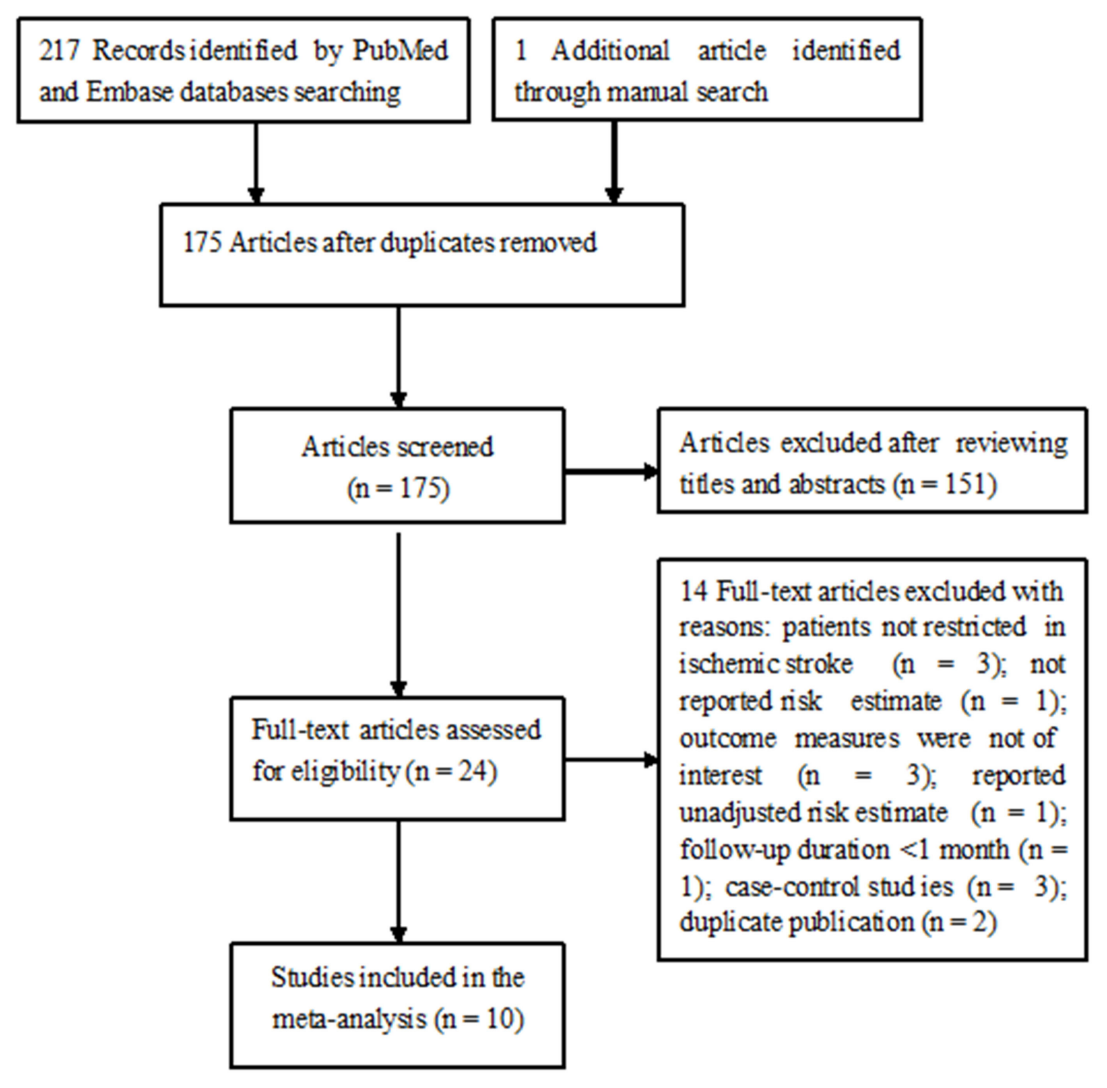
Flow chart of the study selection process.

Figure 1 shows the study selection process. Briefly, a total of 218 potentially relevant articles were identified through electronic and manual searches. After excluding duplicate publications and evaluating titles or abstracts, 24 articles were retrieved for full-text evaluation. In total, 14 articles were further removed after applying the inclusion and exclusion criteria. Thus, 10 studies (13–18, 22–25) were finally included in this meta-analysis.

Table 1 shows a summary of the main characteristics of eligible studies. All eligible studies were conducted in China and published between 2014 and 2020. One study (18) was a *post hoc* analysis of clinical trials and others were prospective cohort studies. The sample sizes of individual studies ranged from 191 to 408, with a total of 2,907 patients. The time of poststroke depression assessment was between 3 months and 1 year. Depression was determined by the Diagnostic and Statistical Manual of Mental Disorders (DSM) and Beck Depression Inventory Fast Screen (BDI-FS) criteria. The prevalence of poststroke depression ranged between 23.3 and 43.5%. According to the NOS, all the included studies were grouped as low risk of bias (NOS 6–8 points).

**Table 1:** Main characteristics of the included studies.

**Study**	**Study design**	**Patients (% men)**	**Age (years)**	**Homocysteine cutoff (mmol/L)**	**PSD diagnosis/** **incidence**	**Follow-up**	**OR (95% CI)**	**Adjusted covariates**	**Total Nos**
Li et al. (13)	P	216 (54.1)	68.9 ± 11.3	Per unit increase	DSM-III-R/43.5%	3 months	1.18 (1.04–1.33)	Age, sex, BMI, stroke syndrome, etiology, NIHSS score, infarct volume, vascular risk factors	7
Li et al. (14)	P	191 (56)	68.5 ± 10.4	Per unit increase	DSM-III-R/41.4%	3 months	1.16 (0.76–1.77)	Age, sex, widowhood, living with offspring, NIHSS score, BMI, Hs-CRP, leptin	7
Tang et al. (15)	P	226 (54.9)	52–81	≥15.5 vs. <15.5; per unit increase	DSM-IV/42.0%	6 months	5.94 (3.07–10.32); 1.13 (1.06–1.23)	Age, sex, BMI, living with offspring, widowhood, vascular risk factors, etiological subtype, infarct volume, and lesion location	7
Li et al. (16)	P	238 (50.0)	55–75	≥16.5 vs. <16.5; per unit increase	DSM-IV/27.3%	3 months	6.65 (3.65–15.21); 1.07 (1.01–1.22)	Age, sex, NIHSS score, BMI, living with offspring, widowhood, education, infarct volume	7
Zhang et al. (17)	P	225 (49.8)	67 (62–73)	Per unit increase	DSM-III-R/32.9%	12 months	1.15 (1.01–1.44)	Age, sex, BMI, stroke syndrome, etiology, NIHSS score, infarct volume, acute treatment, vascular risk factors, mRS at follow-up, history of depression, education, living with offspring, hs-CRP, IGF-1	7
Yin et al. (18)	*Post hoc*	598 (69.6)	60.6 ± 10.3	≥14.65 vs. <14.65	DSM-IV/40.3%	3 months	1.41 (0.91–2.15)	Age, sex, education, BMI, smoking, alcohol, widowhood, time from onset to randomization, SBP, FBG, NIHSS scores, stroke subtype, CHD, DM, hypertension, hyperlipidemia, antihypertensive drugs	8
Cheng et al. (22)	P	259 (49.8)	60 (44–66)	Quartile 4 vs. 1	DSM-IV/36.3%	12 months	3.86 (2.30–7.96)	Age, sex, BMI, etiology, NIHSS score, vascular risk factors, living situation, education, family history of psychiatric disorders, treatment, FBG	8
Li et al. (23)	P	408 (49.8)	65.9 ± 8.6	≥12.36 vs. <12.36	DSM-V/41.2%	3 months	3.84 (2.27–6.50)	NIHSS score, alcohol, platelet to lymphocyte ratio, lesion location, FBG	7
Lu et al. (24)	P	310 (55.5)	62 (51–73)	Per unit increase	BDI-FS/24.5%	3 months	1.07 (1.02–1.11)	Sex, NIHSS score, lesion volumes, education, widowhood or divorced, family history of psychiatric disorders, FBG, CRP, GDF-15, IL-6	7
Zhao et al. (25)	P	236 (60.2)	69 (56–77)	Per unit increase	DSM-IV/23.3%	3 months	1.03 (1.00–1.09)	Age, family history of psychiatric disorders, widowhood or divorced, NIHSS score, Hs-CRP, serum neurofilament light	7

OR, odds ratio; CI, confidence interval; R, retrospective; P, prospective; PSD, poststroke depression; NIHSS, National Institutes of Health Stroke Scale; BMI, body mass index; FBG, fasting blood glucose; CRP, C-reactive protein; Hs-CRP, high-sensitivity C-reactive protein; IL-6, Interleukin 6; GDF-15, growth differentiation factor; IGF-1, insulin-like growth factor 1; mRS, Modified Rankin Scale; DM, diabetes mellitus; CHD, coronary heart disease; SBP, systolic blood pressure; DSM, Diagnostic and Statistical Manual of Mental Disorders; BDI-FS, Beck Depression Inventory Fast Screen; NOS, Newcastle–Ottawa scale.

### Categorical variable analysis of homocysteine level with poststroke depression

**Figure 2:**
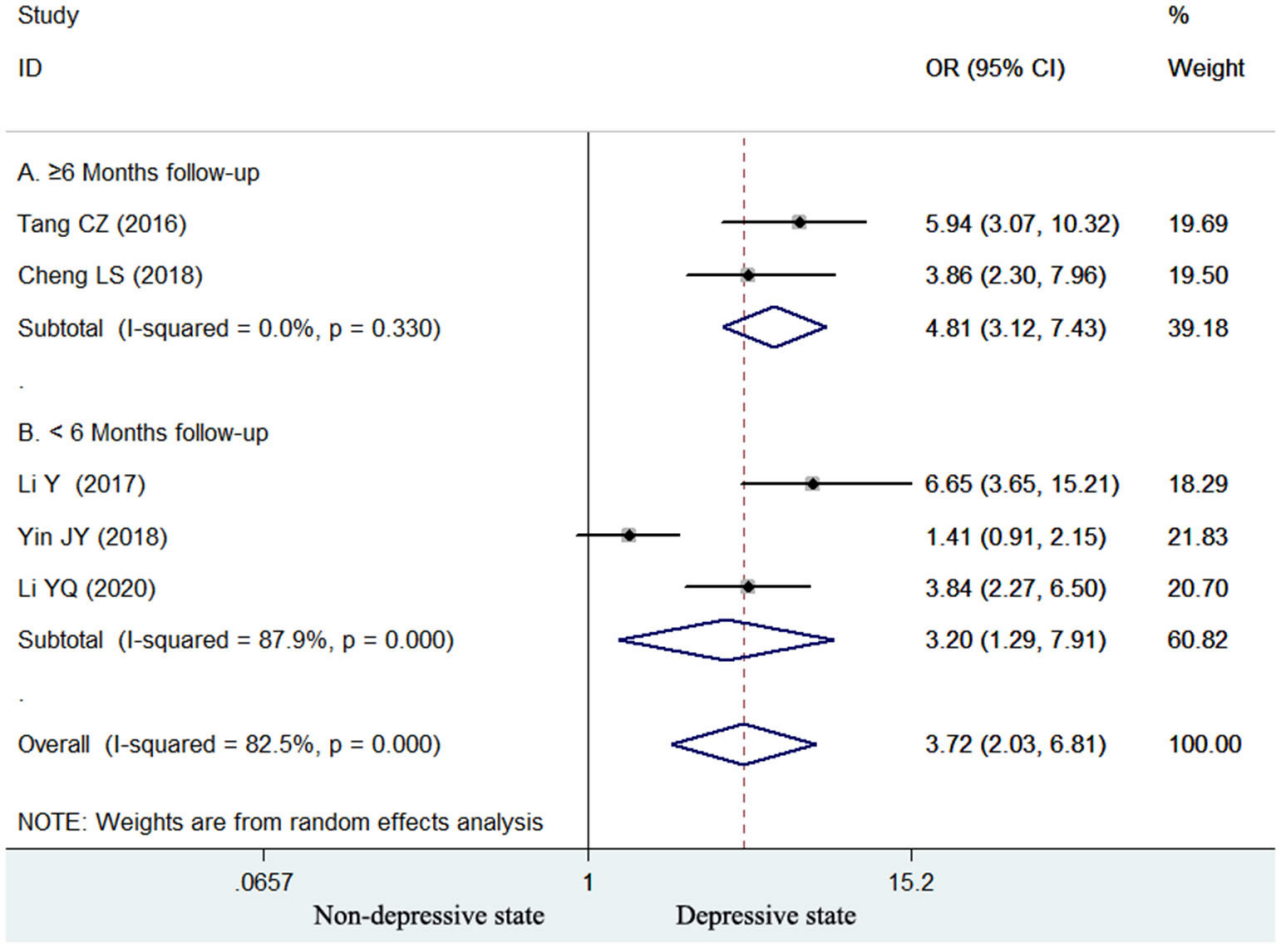
Forest plots showing the pooled OR with 95% CI of poststroke depression for the top vs. bottom homocysteine level in a random effect model.

**Figure 3:**
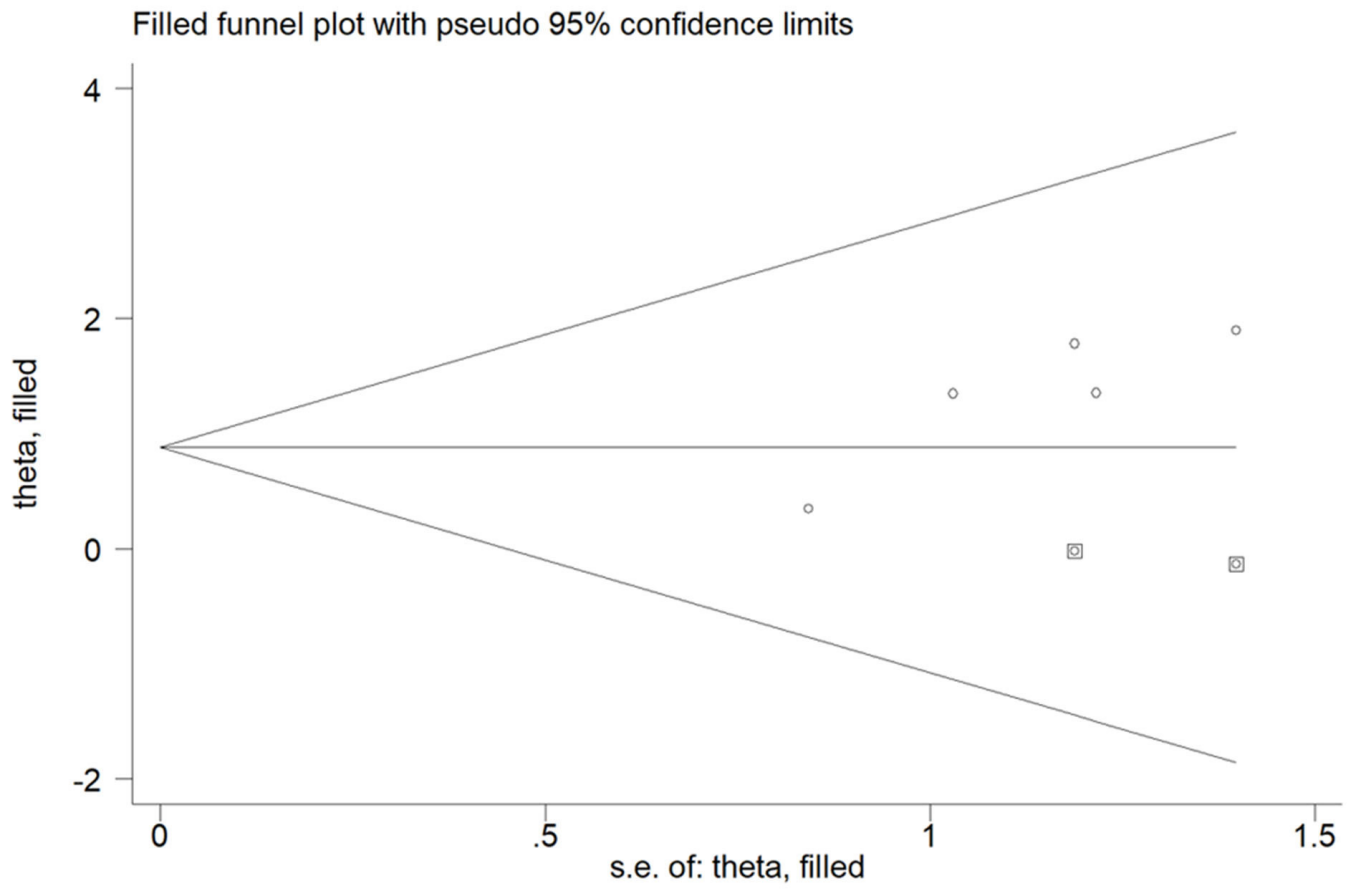
Funnel plot showing the impact of elevated homocysteine level on poststroke depression. The circles alone are real studies and the circles enclosed in boxes are “filled” studies.

Five studies (15, 16, 18, 22, 23) reported the association of elevated homocysteine levels with poststroke depression by categorical analysis. As shown in Figure 2, significant heterogeneity (*I*^2^ = 82.5%, *p* < 0.001) across studies was found. The pooled adjusted OR of poststroke depression was 3.72 (95% CI 2.03–6.81) for the top vs. bottom homocysteine level. Egger's test (*p* = 0.026) but not Begg's test (*p* = 0.462) suggested evidence of publication bias. After imputing two potential missing studies, the pooled OR of poststroke depression (OR 2.41; 95% CI 1.04–5.58) remained statistically significant under a trim-and-fill analysis (Figure 3). Leave-one-out sensitivity analysis demonstrated the robustness of the pooling risk summary (all *p*-values < 0.05; Supplemental Table S1). Subgroup analysis showed that the value of elevated homocysteine level in predicting PSD was stronger in ≥6-month follow-up studies (OR 4.81;95% CI 3.12–7.43; Figure 2A) than those ≤ 3-month follow-up studies (OR 3.20;95% CI 1.29–7.91; Figure 2B).

### Continuous variable analysis of homocysteine level with poststroke depression

**Figure 4:**
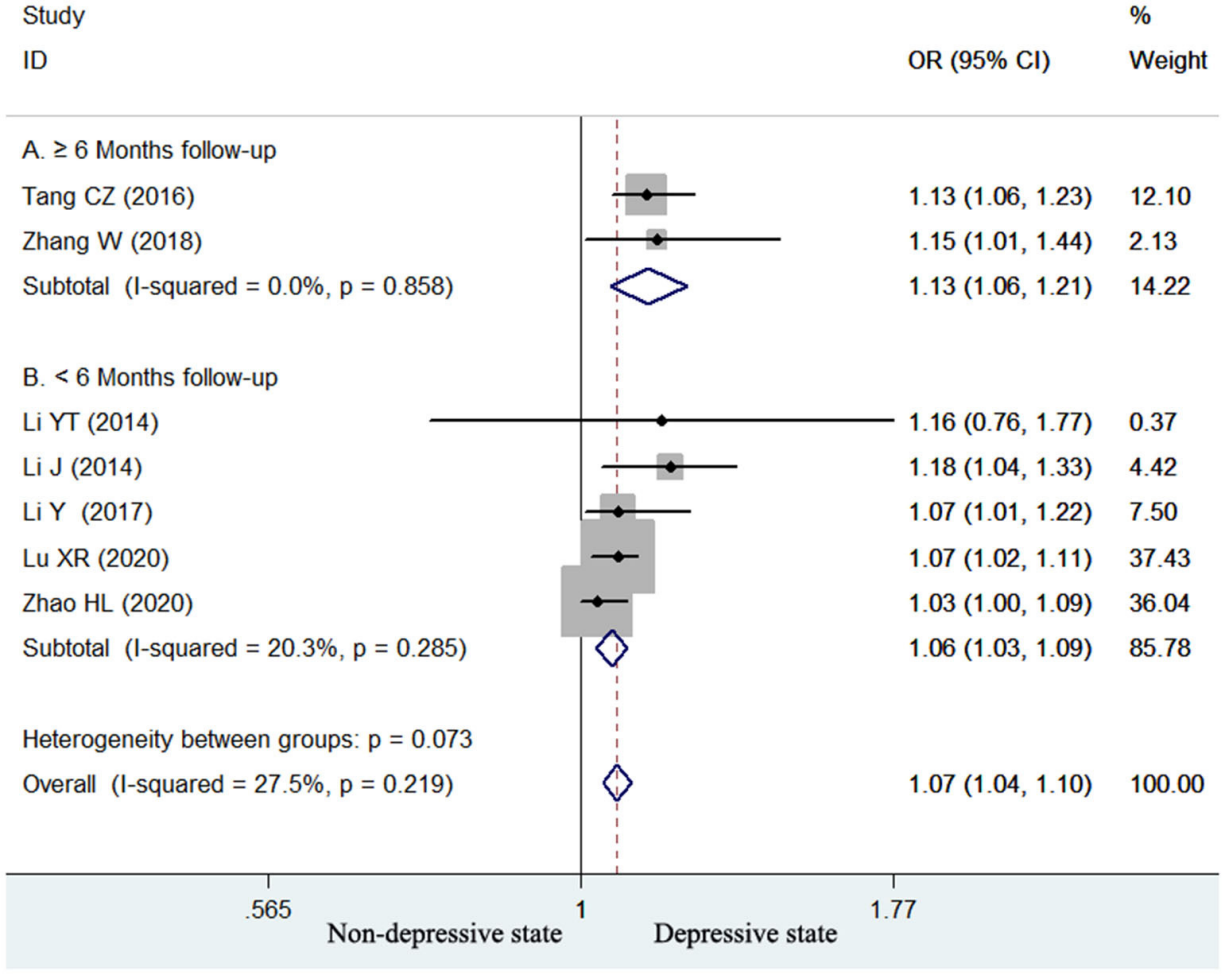
Forest plots showing the pooled OR with 95% CI of poststroke depression for per unit increase in homocysteine level in a fixed-effect model.

Seven studies (13–17, 24, 25) reported the value of homocysteine level in predicting poststroke depression by continuous variable analysis. As shown in Figure 4, no significant heterogeneity (*I*^2^ = 27.5%, *p* = 0.219) across studies was found. The pooled adjusted OR of poststroke depression was 1.07 (95% CI 1.04–1.10) for per unit increase in homocysteine level. Begg's test (*p* = 0.548) and Egger's test (*p* = 0.114) suggested without evidence of publication bias. Leave-one-out sensitivity analysis confirmed the reliability of the pooling risk summary (all *p*-values < 0.05; Supplemental Table S2). Subgroup analysis showed that the value of elevated homocysteine level in predicting poststroke depression was stronger in ≥6-month follow-up studies (OR 1.13; 95% CI 1.06–1.21; Figure 4A) than those ≤ 3-month follow-up studies (OR 1.06; 95% CI 1.03–1.09; Figure 4B).

## Discussion

The current systematic review and meta-analysis analyzed the impact of homocysteine level at the acute stage of ischemic stroke on the development of depression. This meta-analysis demonstrated that acute ischemic stroke patients with elevated homocysteine levels had an increased risk of poststroke depression in stroke survivors. Ischemic stroke survivors with the top homocysteine level had a 3.72-fold higher risk of poststroke depression compared with those with bottom homocysteine levels. Furthermore, a per unit increase in homocysteine level conferred a 7% higher risk of poststroke depression. Blood homocysteine level at the acute stage of ischemic stroke may be an independent predictor of poststroke depression.

A previous meta-analysis (12) has demonstrated that hyperhomocysteinemia had a 34% higher risk of depression in the general population. Our meta-analysis specially focused on the impact of elevated homocysteine levels on depression in patients with acute ischemia. Compared with the general population, the presence of stroke was found to reinforce the predictive value of homocysteine level. A well-designed case-control study (26) showed that stroke survivors (at least 9 months) with major poststroke depression had higher levels of serum homocysteine than those with similar age and functional ability. Another Swedish cohort study (27) demonstrated that elevated homocysteine level was a significant predictor of depressive symptoms among stroke survivors even after accounting for age and gender. These studies further supported that homocysteine may involve in the development of poststroke depression.

The mechanisms underlying elevated homocysteine levels' relationship to the development of poststroke depression have not been fully elucidated. Plausible explanations underlying this association were as follows: (1) elevated homocysteine level can cause neurotransmitter deficiency and inhibit monoamine neurotransmitter metabolism (28); (2) hyperhomocysteinemia can increase the vulnerability of hippocampal neurons to neurotoxic (29, 30) and oxidative injury (31, 32); (3) homocysteine can exaggerate microglia activation and neuroinflammation (33); and (4) homocysteine can upregulate the N-methyl-d-aspartate receptors-mediated synaptic alterations (34).

Depression most frequently developed within the first year after a stroke (35). Subgroup analyses suggested that the value of homocysteine level in predicting poststroke depression appeared to be stronger in ≥6-month follow-up studies than those in ≤ 3-month follow-up studies in both categorical and continuous variable analyses. These findings revealed that the association between elevated homocysteine level and poststroke depression tended to be strengthened with the lengthening of follow-up. Therefore, ischemic stroke patients with elevated homocysteine levels should be early managed with homocysteine-lowering agents.

Depressive symptoms are common among stroke survivors (36). The reported prevalence of poststroke depression ranged between 23.3 and 43.5% in the included studies. Poststroke depression can further increase morbidity and mortality risk. Considering the positive correlation of hyperhomocysteinemia with poststroke depression, the determination of blood homocysteine level can provide early prognostic information. On the other hand, whether management of hyperhomocysteinemia can reduce depression risk among stroke survivors is another interesting issue. However, a randomized controlled trial showed that lowering homocysteine levels by supplementation with vitamin B_12_ and folic acid did not reduce depressive symptoms in older adults with hyperhomocysteinemia (37). Future clinical trials are warranted to investigate the impact of the homocysteine-lowering intervention on the development of poststroke depression.

Several limitations should be acknowledged in our study. First, blood homocysteine level was tested only at the acute stage of stroke rather than dynamic measurement. Single detection of homocysteine level at a time point may have led to selection bias. Second, various thresholds of elevated homocysteine levels were reported in the analyzed studies, thus preventing clinicians from discriminating against those in need of homocysteine-lowering intervention. Third, significant heterogeneity (*I*^2^ = 82.5%) was present in the pooling risk summary by categorial homocysteine analysis. Different thresholds of elevated homocysteine level, time of depression assessment, and methods of depression diagnosis may contribute to the heterogeneity. Fourth, the lack of adjusting several uncontrolled confounding factors such as folate, vitamin B6, or vitamin B_12_ level may confound the pooling risk estimate. Fifth, all included patients were from the Chinese population and generalization of the current results to other patients should be done with caution. Finally, this meta-analysis could not distinguish the impacts of elevated homocysteine levels on different types of depression and subtypes of ischemic stroke.

## Conclusion

Elevated homocysteine level at the acute stage of ischemic stroke may be an independent predictor of poststroke depression in stroke survivors. Future studies are necessary to investigate whether homocysteine-lowering intervention can reduce the development of poststroke depression.

## Data availability statement

The original contributions presented in the study are included in the article/Supplementary material, further inquiries can be directed to the corresponding author.

## Author contributions

Study conception, design, and revising the article critically for important intellectual content: JZ. Literature search, acquisition of data, data extraction, and interpretation of data: YLia and XS. Data analysis: LC and YLi. Drafting the article: YLia. All authors have read the final approval of the version to be published.
