# Supplementary material for: Homocysteine level at the acute stage of ischemic stroke as a biomarker of poststroke depression: A systematic review and meta-analysis
Source: Front Psychiatry. 2023 Feb 13;13:1016700. doi: 10.3389/fpsyt.2022.1016700 (PMC9968726; doi:10.3389/fpsyt.2022.1016700)
Supplement: Supplementary file 2 [file Table_2.DOC]

~~Supplemental table S1 Results of sensitivity analysis on categorical variable analysis of CRP level~~

| Removal of single study | Pooled risk ratio | 95% confidence intervals | Heterogeneity between studies |
| --- | --- | --- | --- |
| Tang CZ 2016  Yin JY 2018  Li YQ 2020  Cheng LS 2018  Li Y 2017 | 3.32  4.73  3.73  3.72  3.27 | 1.68–6.57  3.50–6.41  1.69–8.21  1.74–7.94  1.69–6.33 | *p*<0.001; I^2^ =83.2%  *p*=0.488; I^2^ =0.0%  *p*<0.001; I^2^ =86.5%  *p*<0.001; I^2^ =86.6%  *p*<0.001; I^2^ =83.4% |

~~Supplemental table S2 Results of sensitivity analysis on continuous variable analysis of CRP level~~

| Removal of single study | Pooled risk ratio | 95% confidence intervals | Heterogeneity between studies |
| --- | --- | --- | --- |
| Tang CZ 2016  Li YT 2014  Zhang W 2018  Lu XR 2020  Zhao HL 2020  Li J 2014  Li Y 2017 | 1.06  1.07  1.07  1.07  1.09  1.06  1.07 | 1.03–1.09  1.04–1.10  1.04–1.10  1.03–1.10  1.06–1.13  1.04–1.09  1.04–1.10 | *p*=0.332; I^2^ =14.3%  *p*=0.149; I^2^ =38.5%  *p*=0.179; I^2^ =14.3%  *p*=0.142; I^2^ =39.5%  *p*=0.557; I^2^ =0.0%  *p*=0.339; I^2^ =11.9%  *p*=0.142; I^2^ =39.6% |
